# FocusedON-BC: A Robust Deep Learning Framework for Automated Body Composition Assessment

**DOI:** 10.3390/nu18091477

**Published:** 2026-05-06

**Authors:** Jano Manuel Rubio-García, Andrés Jiménez-Sánchez, Fiorella Palmas, Cora Oliver-Vila, Aitor Rodriguez-Martinez, Nuria Roson-Gradaille, Selenia Maria Medina-Hernandez, Gabriel Santana-Quintana, Eduardo J. Carrasco, Raul Guerra, Rosa Burgos, Andreea Ciudin

**Affiliations:** 1 Department of Radiology, Complejo Hospitalario Universitario Insular Materno Infantil de Canarias, 35016 Las Palmas de Gran Canaria, Spain; 2 Endocrinología y Nutrición, Instituto de Biomedicina de Sevilla, IBiS/Hospital Universitario Virgen del Rocío/CSIC/Universidad de Sevilla, Avda. Manuel Siurot s/n, 41013 Seville, Spain; 3 Endocrinology and Nutrition Department, Hospital Universitari Vall d’Hebron, 08035 Barcelona, Spain; rosa.burgos@vallhebron.cat (R.B.); andreea.ciudin@vallhebron.cat (A.C.); 4 Diabetes and Metabolism Research Unit, Vall d’Hebron Institut de Recerca (VHIR), 08035 Barcelona, Spain; 5 Department of Radiology, Institut de Diagnòstic per la Imatge (IDI), Hospital Universitari Vall d’Hebron, 08035 Barcelona, Spain; 6 ARTIS Development, 35017 Las Palmas de Gran Canaria, Spainrguerra@artisdevelopment.com (R.G.); 7 Department of Medicine, Universitat Autònoma de Barcelona, 08193 Barcelona, Spain; 8 Centro de Investigación Biomédica en Red de Diabetes y Enfermedades Metabólicas Asociadas (CIBERDEM), Instituto de Salud Carlos III (ISCIII), 28029 Madrid, Spain

**Keywords:** body composition, computed tomography, deep learning, opportunistic screening

## Abstract

Background: Computed tomography-based body composition assessment enables the quantification of clinically relevant prognostic conditions such as sarcopenia, myosteatosis, and visceral adiposity; the manual segmentation process limits its routine implementation in clinical practice. We developed FocusedON-BC, an automated deep learning tool for opportunistic screening of skeletal muscle (SM), visceral adipose tissue (VAT), and subcutaneous adipose tissue (SAT) across the T12–L5 range; Methods: Validated on a multicenter cohort of 518 patients (3280 slices) with diverse body mass index (12.7–47.7 kg/m^2^) from different computed tomography manufacturers. Performance was benchmarked against expert segmentation using the Dice coefficient score (DSC) and the mean absolute error (MAE); Results: FocusedON-BC achieved expert-level accuracy: mean DSC was 0.974±0.010 (SM), 0.959±0.032 (VAT), and 0.986±0.014 (SAT). Clinical MAE remained <5% for all compartments. Performance was robust, independent of body mass index and computed tomography scanner model. Qualitative assessment confirmed the tool’s capability to isolate intermuscular adipose tissue for radiodensity analysis; Conclusions: FocusedON-BC provides accurate, vendor-agnostic body composition and muscle quality analysis. Its reliability across diverse phenotypes supports implementation for routine nutritional screening.

## 1. Introduction

Body composition has emerged as a key determinant of clinical prognosis across a wide range of chronic and oncological diseases [1]. Reduced skeletal muscle mass and area, frequently used as radiological surrogates of sarcopenia (e.g., defined by a Skeletal Muscle Index $<39.0\;{\mathrm{cm}}^{2}/m^{2}$ for women and <55.0 ${\mathrm{cm}}^{2}/m^{2}$ for men), as well as increased visceral adiposity (e.g., VAT area $>130\;{\mathrm{cm}}^{2}$), are independently associated with lower survival, greater treatment-related toxicity, and a higher risk of postoperative complications, including surgical site infections, delayed wound healing, and prolonged hospital stays [2,3]. In addition, muscle quality (assessed through radiological attenuation and intramuscular fat infiltration or myosteatosis) provides prognostic information beyond that offered by body mass index (BMI, calculated as $\mathrm{weight}\;\left(\mathrm{kg}\right)/\mathrm{height}\;{\left(m\right)}^{2}$) alone [4,5,6]. Consistent associations have been found between these computed tomography (CT)-derived muscle and adipose tissue phenotypes and clinically relevant outcomes, including postoperative complications and mortality [5,6,7]. This evidence supports the use of CT-based body composition analysis in risk stratification and nutritional assessment.

Despite this growing body of evidence, the systematic quantification of these imaging biomarkers remains uncommon in routine clinical practice [8]. The reference standard for body composition assessment in clinical settings is the analysis of CT images, most frequently at the third lumbar vertebra (L3) [9]. This specific anatomical level is the universally accepted benchmark because tissue cross-sectional areas at L3 have been shown to highly correlate with total-body skeletal muscle and adipose tissue volumes. Usually, this quantification has relied on the definitions established by the Alberta Protocol [10], which standardizes tissue segmentation based on specific Hounsfield Unit (HU) ranges. However, accurate manual segmentation is a labor-intensive and operator-dependent process that can take 15–20 min per patient and requires specialized training [11,12]. This bottleneck restricts body composition analysis to small research cohorts, preventing the opportunistic screening for body composition alterations in millions of patients who undergo abdominal CT scans annually for diagnostic purposes [8]. Additionally, manual assessments that rely exclusively on radiodensity thresholds rather than strict anatomical boundaries may be inadequate for automated systems. This is particularly critical when defining the true boundaries of the skeletal muscle (SM) compartment, where the intensity of pixels often overlaps with the surrounding internal organs, leading to potential overestimation of the muscle area [13,14].

To address this limitation, semi-automated segmentation software (e.g., SliceOmatic, Tissue Compass, OsiriX, etc.) was introduced [15,16] to accelerate segmentation but still requires substantial human intervention to correct errors. To further optimize both workflow and output, interest has recently shifted towards deep learning frameworks [17,18]. While comparative analyses [19] indicate that these deep learning-based models have achieved high segmentation accuracy across various cohorts, the transition from research prototypes to fully integrated clinical tools remains a challenge, necessitating robust, CT vendor-agnostic solutions capable of handling diverse acquisition protocols [17,18].

Tackling these unmet needs, we evaluated FocusedON-BC (ARTIS Development, Spain), a proprietary automated deep learning system designed for a user-friendly, high output, and opportunistic assessment of body composition using CT images. The system utilizes a dual-model architecture to precisely identify the abdominal region (excluding air) and subsequently segment into SM, visceral adipose tissue (VAT), and subcutaneous adipose tissue (SAT). Unlike studies focused on incremental architectural optimizations or component-level algorithmic benchmarking, the present work centers on the rigorous clinical validation of a production-ready, locked medical software framework. The objective of this study was to rigorously validate the clinical performance of FocusedON-BC on a large, independent, and multicenter cohort. Specifically, we aimed to demonstrate that the algorithm achieves expert-level geometric accuracy, targeting a Dice similarity coefficient (DSC) >0.90, consistent with current deep learning benchmarks [12,19], and high quantitative accuracy. In addition, we established a strict mean absolute error (MAE) goal of <5% to ensure performance remains within the typical range of inter-observer variability reported in manual segmentation studies [12,14].

## 2. Materials and Methods

The methodology of this study was designed to develop a deep learning framework capable of generalization across different clinical scenarios and technical conditions. The methodology is structured into four key phases: (1) curation of diverse clinical datasets to ensure variability; (2) manual generation of the ground truth by trained personnel following a strict anatomical protocol; (3) development and training of the FocusedON-BC architecture; and (4) statistical validation of the tool against standard references.

### 2.1. Study Design

A retrospective multicentric study was designed to ensure the developed tool functions robustly across a variety of clinical scenarios. The study incorporates CT images from distinct sources divided into two evaluation phases: a primary clinical cohort retrospectively collected at the Endocrinology and Nutrition Department of Vall d’Hebron University Hospital (Barcelona, Spain) (dataset 1) and a publicly available repository (dataset 2) used together for cross-validation, and an aggregate of four public collections for the independent hold-out test (dataset 3).

Dataset 1 corresponds to the FLASH Study and includes 270 patients. The study was approved by the local Ethics Committee (PR(AG)361/2025) and carried out in accordance with the Declaration of Helsinki. All the patients signed the informed consent form before participating in the study. Dataset 2 comprises 248 patients extracted from the SAROS public dataset [20] obtained from The Cancer Imaging Archive (TCIA). The SAROS dataset comprises images aggregated from multiple collections within TCIA, representing a predominantly North American (United States and Canada) patient population.

The independent hold-out test cohort (N=70) was established as Dataset 3. To ensure a highly heterogeneous, multi-institutional, and international sample, this dataset was compiled by aggregating completely anonymized and de-identified clinical images from four independent public collections from TCIA: TCGA-ESCA [21], ACRIN-FLT-Breast [22], VAREPOP-APOLLO [23], and ACRIN-NSCLC-FDG-PET [24]. This combined dataset represents a predominantly North American patient population, along with a small percentage of patients from South Korea.

The inclusion criteria for both datasets involve adult patients (age >18 years) with available abdominal CT scans covering the T12–L5 vertebral range. Exclusion criteria included CT images with significant artifacts, such as severe metallic implants or severe anatomical distortions, which were dynamically excluded during the initial visual screening prior to annotation (exact exclusion counts were not systematically recorded). Demographic metadata, including age, sex, and BMI, were retrieved to characterize the study population. To ensure real-world representativeness and specifically evaluate the algorithm’s robustness against the technical variability (e.g., variations in noise and texture profiles) introduced by different acquisition hardware, the cohort deliberately included images from different CT manufacturers.

### 2.2. Manual Segmentation and Ground Truth Generation

**Figure 1:**
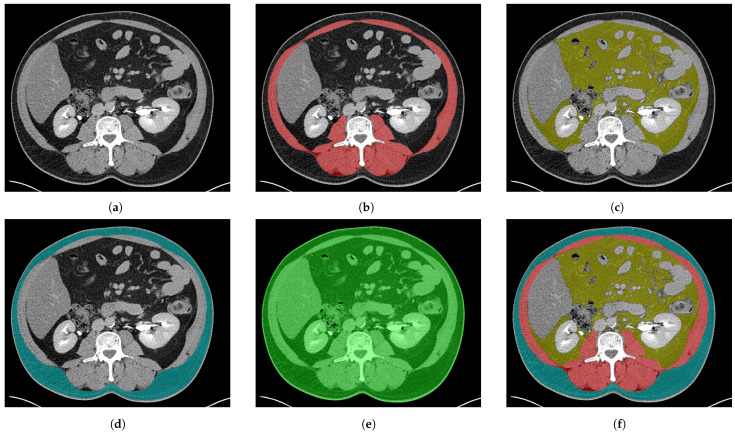
Ground truth examples of each class using the proposed protocol. (**a**) Original CT slice; (**b**) skeletal muscle (SM) mask (red); (**c**) visceral adipose tissue (VAT) mask (yellow); (**d**) subcutaneous adipose Tissue (SAT) mask (blue); (**e**) body region mask (excluding air) (green); (**f**) combined ground truth mask showing all tissue compartments.

The manual annotation of the ground truth was performed using FocusedON-BC (Research version v2.1, ARTIS Development, Las Palmas de Gran Canaria, Spain) by trained personnel under the supervision of senior medical specialists with advanced expertise in CT-based body composition assessment (co-authors of this study). This ground truth serves as the clinical gold standard for algorithm training and evaluation. The annotation process was based on a strict anatomical protocol defined by these experts to ensure replicability. This protocol followed an approach based on fascia identification, representing a methodological refinement of the standard Alberta Protocol. The deep fascia refers to the macroscopic fascial structures delineating the skeletal muscle compartment, including the thoracolumbar fascia posteriorly and the abdominal wall fascia anteriorly and laterally. While the classic protocol often relies on semi-automated thresholding of all pixels within the abdominal cross-section, the proposed protocol prioritizes the definition of the anatomical container. Following this protocol, CT series were segmented (Figure 1) into distinct classes: SM, VAT, SAT, and the body region mask.

Finally, a spatially sparse sampling strategy was applied to prioritize the informational value of each training sample. Instead of annotating full volumetric blocks where adjacent slices provide minimal new information, the ground truth was restricted to representative axial slices at specific anatomical landmarks within the T12–L5 range. This strategy mitigates the risk of overfitting to repetitive volumetric patterns, a common source of bias where models essentially memorize highly correlated, adjacent slices from the same patient rather than learning generalizable tissue boundaries, which can artificially boost segmentation metrics without improving real-world performance. Although this sparse approach makes the segmentation task significantly more demanding for the algorithm, it guarantees that the resulting model is robust and capable of handling diverse anatomical presentations. All masks underwent a dual-verification process: an automated technical check for metadata consistency, followed by a manual refinement and final approval by trained experts.

#### 2.2.1. Muscle Tissue

The SM compartment (Figure 1b) was manually segmented across the abdominal, lower thoracic, and upper pelvic regions (vertebral levels from T12 to L5), encompassing the paravertebral, abdominal wall, psoas, quadratus lumborum, intercostal, and gluteal muscles. A key divergence from the standard Alberta protocol lies in our anatomical definition of the muscle boundary: while the Alberta protocol typically excludes non-contractile tissues such as tendons and ligaments using radiodensity thresholds, the proposed protocol prioritizes structural continuity to enhance inter-observer replicability. Consequently, connective structures such as intramuscular tendons and intercostal cartilage were explicitly retained within the SM compartment. Although non-contractile, their inclusion ensures that the deep learning model learns a deterministic boundary, thereby avoiding the high variability associated with manually tracing irregular cartilage interfaces. This deterministic, anatomy-driven approach was specifically designed to minimize inter- and intra-observer variability a priori, guaranteeing a highly reproducible ground truth across different annotators and precluding the need for a post-hoc variability study.

#### 2.2.2. Adipose Tissue

Adipose tissue compartments were differentiated based on their anatomical location relative to the abdominal wall.

VAT class (Figure 1c) was defined as all adipose tissue located within the abdominal cavity, encompassing both intraperitoneal (mesenteric and omental) and retroperitoneal depots surrounding the abdominal organs. A critical refinement in our protocol compared to standard threshold-based methods was the rigorous exclusion of ectopic adipose tissue, such as that contained within solid organs (e.g., hepatic steatosis or pararenal adipose tissue), and bone marrow was explicitly masked. This ensures that the metric strictly represents the intra-abdominal, inter-organ adipose depot, avoiding overestimation of VAT in patients with significant organ steatosis.

The SAT class (Figure 1d) was defined as the adipose layer located between the skin and the outer abdominal muscle fascia. Deviating from protocols that rely solely on external contour thresholding, our methodology enforced the strict exclusion of the skin (dermis and epidermis) from the SAT volume. This anatomical subtraction is essential to prevent overestimation of adiposity, particularly in patients with low body fat (BMI < $18.5\;{\mathrm{kg}/m}^{2}$), where skin thickness represents a confounding proportion of the external layer.

#### 2.2.3. Body Region

To complete the semantic map, the body region mask (Figure 1e) was defined as the entire area occupied by the patient’s abdominal region in the CT images. This included all anatomical structures within this region, excluding external elements or any other artifacts. In addition, internal air cavities, such as bowel gas and gastric air, were excluded from this class to prevent variability in tissue area calculations caused by transient factors like gastrointestinal gas content or respiratory phase.

### 2.3. FocusedON-BC Architecture

**Figure 2:**
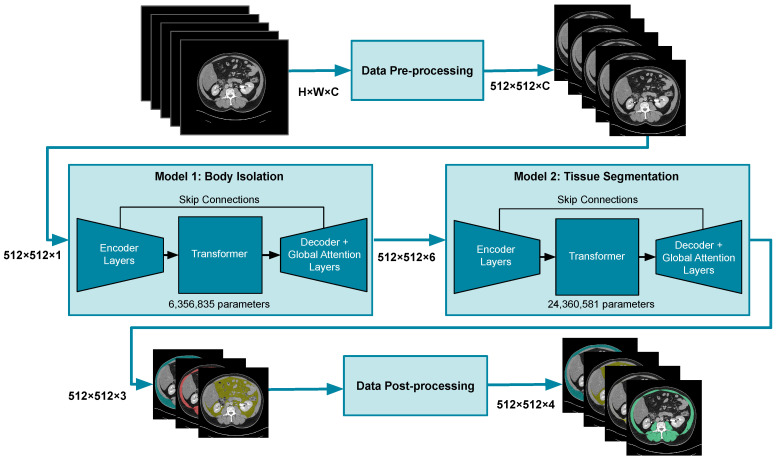
Schematic representation of the FocusedON-BC architecture. The workflow comprises data pre-processing, body isolation (Model 1), and multi-tissue segmentation (Model 2) using a hybrid Transformer-U-Net structure. The diagram specifies the encoder/decoder layers, attention layers, transformer blocks, skip connections, and the total parameter count for each stage. Stacked image represent input and output, where the overlap is intended to symbolize the volumetric nature of the CT data or the multiples outputs mask.

The FocusedON-BC framework was designed to automatically quantify body composition from abdominal CT images. The framework implements a sequential cascade pipeline consisting of data standardization, a dual-model inference engine, and a deterministic post-processing stage for tissue fractionation. The dual model was implemented with two specific objectives: (i) body identification and (ii) tissue segmentation. The detailed architecture of the FocusedON-BC framework is illustrated in Figure 2. The pipeline consists of two primary stages: a body isolation module (Model 1, 6.3 M parameters) and a tissue segmentation module (Model 2, 24.3 M parameters). Model 2 employs a hybrid architecture that integrates the spatial precision of a U-Net with the global context capabilities of Transformer blocks and global attention layers. As shown in the Figure 2, the encoder extracts hierarchical features, while the Transformer layer at the bottleneck captures long-range dependencies, which are then integrated through skip connections into the decoder layers for the final multi-class segmentation (SM, VAT, and SAT).

#### 2.3.1. Data Pre-Processing

Prior to the incorporation of data into the neural networks, a preprocessing pipeline was applied to all CT slices. First, the volumetric data were resampled to enforce a fixed slice spacing of 3.0 mm using a vertical interpolation. After that, a spatial resizing of each axial slice was applied to reach a fixed matrix dimension of 512 × 512 pixels. Finally, all pixel intensities were clipped to the specified Hounsfield Unit windows and linearly rescaled to a [0, 1] range to generate the standardized input tensors required by the neural network architecture.

#### 2.3.2. Network Architecture

The architecture is based on two specialized neural networks sharing a Hybrid Transformer-UNet topology. The first stage is body isolation, specifically designed to identify the abdominal body across the T12–L5 range, regardless of vertebral level. This volumetric coverage ensures the system is not restricted to specific anatomical landmarks (e.g., L3) but can process any axial slice within this range, providing flexibility when standard levels are compromised by artifacts. This network operates on a single axial slice standardized to a wide intensity window (−400 to 200 HU). This maximizes the contrast between the body contour and the background and generates a binary mask that excludes external artifacts and intraluminal air cavities, ensuring the correct delineation of the body region.

Subsequently, the mask of the body region serves as input to the second network (tissue segmentation stage). Unlike the first step, this network is optimized to analyze a stack of five consecutive slices simultaneously (acting as a multi-channel 2.5D input), allowing the model to capture longitudinal anatomical continuity. This network is trained to classify pixels into anatomical compartments (VAT, SAT, SM) based on spatial features and fascial boundaries, utilizing a soft-tissue window (−150 to 200 HU).

#### 2.3.3. Data Post-Processing

To transform the output of the SM class into lean muscle and intermuscular adipose tissue (IMAT) classes, a post-processing stage was applied. Unlike VAT and SAT, which are predicted directly by the semantic segmentation model, IMAT quantification follows a hybrid anatomical-radiometric approach. FocusedON-BC framework first segments the total SM compartment defined by the deep fascia. Before quantification, a morphological smoothing filter is applied to the raw mask to eliminate pixel noise and enforce structural consistency. Subsequently, a standardized radiodensity analysis is performed strictly within this anatomical mask. Strictly adhering to clinically standardized and biologically defined radiological guidelines, pixels with attenuation values below −30 HU are classified as IMAT, while those above are retained as lean muscle [10]. This fascia-guided strategy ensures that myosteatosis is accurately differentiated from lean tissue and isolated from surrounding extramuscular adiposity.

#### 2.3.4. Training and Evaluation Strategy

To ensure both the fundamental stability of the neural network and its real-world generalizability, we implemented a two-phase evaluation strategy: K-fold validation and independent testing.

First, a 10-fold cross-validation approach was employed on the primary design cohort of 518 patients (3280 slices). The dataset was partitioned into ten parts strictly at the patient level to prevent data leakage. In clinical machine learning, this cross-validation phase is paramount for demonstrating methodological robustness. By training and evaluating the model across 10 independent iterations (utilizing a 90% training and 10% validation split, with distinct random weight initializations and disjoint validation folds) we ensure that the architecture and loss function converge systematically. This process shows that high performance is not an artifact of accidental, lucky initialization or a particularly favorable data split, but rather a fundamentally sound algorithmic design that does not overfit. During this phase, a data augmentation strategy was applied exclusively to the training partitions, including elastic deformations and simulated noise profiles, to mimic the characteristics of different CT scanner manufacturers and further enhance vendor neutrality.

Once the architecture’s stability was verified through the K-fold process, a final production candidate model was trained utilizing the entire augmented primary cohort to maximize anatomical feature exposure. Given the robustness of the methodology, as demonstrated on smaller subsets during cross-validation, training the identical architecture on the full dataset minimizes the risk of overfitting.

Finally, to definitively confirm real-world clinical safety, this production model was evaluated on a strictly isolated and independent hold-out test dataset comprising 70 unique patients (138 slices). This cohort was functionally locked out of all prior pipelines. This two-tiered strategy guarantees that the reported metrics reflect true clinical generalizability rather than an overestimation of the model’s capabilities.

Model training and computational experiments were conducted on a high-performance workstation equipped with NVIDIA® RTX™ 5000 ADA Generation GPUs (NVIDIA Corporation, Santa Clara, CA, USA) with 32 GB total VRAM and an AMD Ryzen™ Threadripper™ PRO 7975WX CPU (Advanced Micro Devices, Inc., Santa Clara, CA, USA), running on an Ubuntu 22.04 environment (Canonical Ltd., London, UK).

### 2.4. Statistical Analysis and Performance Metrics

Statistical analysis was performed using Python version 3.11.2 (Python Software Foundation, Wilmington, DE, USA) with the SciPy Stats package version 1.16.3 (SciPy Community, Austin, TX, USA). Continuous variables are expressed as mean ± standard deviation (SD) or median with interquartile range (IQR). Categorical variables are presented as frequencies and percentages. The performance of the models was evaluated using different evaluation metrics. To analyze geometric segmentation performance, the DSC was employed. The DSC (Equation (1)) measures the spatial overlap between the algorithmic prediction ($Y_{pred}$) and the expert ground truth ($Y_{true}$) in a range between 0 and 1 (where 1 indicates perfect anatomical alignment). However, to assess clinical measurement reliability, the Mean Absolute Error (MAE) was also calculated. MAE measures the average magnitude of errors between the predicted tissue area ($A_{pred}$) and the true tissue area ($A_{true}$) in absolute terms (${\mathrm{cm}}^{2}$) across *N* evaluated slices, and is defined in Equation (2). We utilized MAE because it provides a direct, highly interpretable clinical metric of the real-world volume deviation a physician would encounter, independent of the spatial overlap. In addition, performance was stratified by BMI categories (underweight to obesity), age groups, and scanner manufacturer to verify robustness.


(1) $$\mathrm{DSC}=\frac{2|Y_{\mathrm{pred}}\cap Y_{\mathrm{true}}|}{|Y_{\mathrm{pred}}\left|+\right|Y_{\mathrm{true}}|},$$

(2) $$\mathrm{MAE}=\frac{1}{N}\sum_{i=1}^{N}\left|A_{\mathrm{pred},i}-A_{\mathrm{true},i}\right|$$

## 3. Results

The clinical validation of the FocusedON-BC framework was conducted in a total study population of 588 patients through a two-phase evaluation strategy. First, a 10-fold cross-validation was performed on the primary cohort of 518 patients (3280 slices) to ensure robust internal validation and comprehensive subgroup analysis on unseen data. Second, a strictly isolated hold-out dataset of 70 unique patients (138 slices) was utilized to independently confirm the real-world generalizability of the production-ready model. The quantitative analysis is presented below, stratified by geometric accuracy (the spatial and morphological overlap between the prediction and the ground truth, measured via DSC) and clinical measurement precision (the error in total computed tissue area) for both validation phases. In addition, the performance analysis for the musculoskeletal compartment focused exclusively on the aggregate SM class (defined by the deep fascia). Since the differentiation between lean muscle and IMAT is performed via a deterministic radiodensity thresholding within this boundary, the geometric accuracy of the fascial container serves as the validating proxy for both sub-compartments.

### 3.1. Study Population

The study included a total population of 588 unique patients and an analysis of 3418 CT slices. This sample was divided into two distinct groups based on the validation strategy (Table 1).

The validation cohort comprised 518 unique patients, corresponding to a total of 3280 analyzed CT slices. The validation population exhibited a heterogeneous demographic profile, with 47.7% (247) male and 37.6% (195) female participants (sex data were unavailable for the remaining 14.7% of cases). The mean age was 60.8±14.2 years (range: 19–92 years), ensuring representation from young adults to geriatric subgroups. Regarding nutritional status, the cohort covered a wide spectrum of body compositions, with BMI values ranging from $12.7\;{\mathrm{kg}/m}^{2}$ (underweight) to $47.7\;{\mathrm{kg}/m}^{2}$ (obesity). To assess technical generalizability, images were acquired from different scanner manufacturers, predominantly Siemens Healthineers (56.4%), followed by Philips (12.5%), GE Healthcare (10.2%), and Toshiba Medical Systems (9.3%). In addition, the images were collected from 50 different scanner models, including high-performance systems such as the Siemens SOMATOM Force (Siemens Healthineers, Erlangen, Germany) and Philips iCT 256 (Philips, Amsterdam, The Netherlands), as well as widely used platforms like the GE LightSpeed (GE Medical Systems, Chicago, IL, USA) and Siemens Sensation series (Erlangen, Germany). On the other hand, the independent test cohort (hold-out test cohort in Table 1) included 70 additional patients (138 slices) with a mean age of 63.4±11.9 years, a BMI range of 16.9 to $49.0\;{\mathrm{kg}/m}^{2}$, and balanced biological distribution (60% male). This test set includes 16 unique scanner models from three major manufacturers (Siemens, GE, and Toshiba). Notably, it incorporates specialized imaging systems such as the Siemens Biograph and Emotion series, which are widely used in oncological and clinical research. Collectively, the complete study population included patients referred for oncological assessment, encompassing a variety of pathologies such as pancreatic cancer, kidney cancer, and sarcomas, ensuring a consistent clinical context across the multicenter cohort. The dataset was specifically curated to reflect uncurated clinical variability, encompassing images from both standalone CT and PET-CT systems. The acquisition techniques varied widely in terms of tube voltage (kVp), current (mA), and reconstruction kernels. Furthermore, examinations included non-contrast scans, oral contrast protocols, and intravenous contrast-enhanced scans acquired at multiple timings (primarily portal-venous, but also arterial and urographic phases), confirming the tool’s adaptability to routine clinical practice.

### 3.2. Segmentation Performance

The performance of the FocusedON-BC framework was evaluated using a 10-Fold Cross-Validation strategy applied to the entire study cohort. For all evaluated cases, segmentation accuracy was quantified computationally using the DSC by comparing the automated predictions against their corresponding expert-verified ground truth masks. To ensure an unbiased estimation of clinical performance, Table 2 summarizes the results generated from the validation folds.

The framework demonstrated high segmentation accuracy across both stages of the pipeline. First, the body identification stage achieved a DSC>0.999 regardless of the anatomical level. Subsequently, the tissue segmentation stage demonstrated high stability across the entire anatomical range. The performance at the anatomical boundaries (T12 and L5) showed clinically acceptable performance compared to the standard L3 landmark. The SM class achieved a global DSC of 0.974±0.010, peaking at L3 (0.979±0.009). In addition, SAT yielded the highest global results (0.986±0.014), with a maximum DSC observed at L5 (0.990±0.008). Finally, despite the morphological complexity of VAT, due to bowel peristalsis and heterogeneous texture, it maintained a robust global mean DSC of 0.959±0.033, with consistent DSC across all levels.

**Figure 3:**
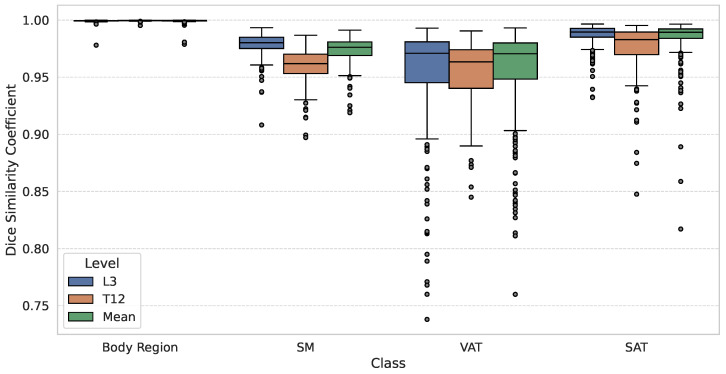
Comparative boxplots of dice similarity coefficients (DSC) for body region, skeletal muscle (SM), visceral adipose tissue (VAT), and subcutaneous adipose tissue (SAT). The distribution is stratified by anatomical level: L3 (standard landmark), T12 (thoracic boundary), and the global mean across the different landmarks segmented. Boxplots show medians (center line), IQR (box), whiskers (1.5 × IQR), and outliers (circles).

**Figure 4:**
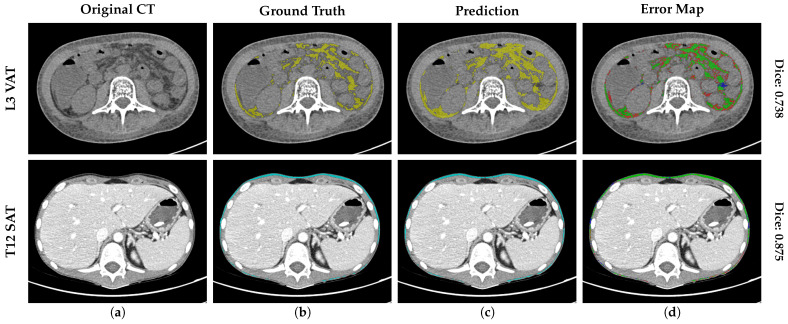
Analysis of worst-case scenarios of the validation cohort. Segmentation results for the outliers with the lowest geometric accuracy (DSC < 0.90). (**a**) The original CT image, (**b**) the ground truth mask (VAT in yellow and SAT in blue), (**c**) the Prediction (VAT in yellow and SAT in blue), and (**d**) the error map highlighting discrepancies, where green pixels represent the correct intersection (true positives), red pixels indicate over-segmentation (false positives), and blue pixels denote under-segmentation (false negatives).

Figure 3 visualizes the DSC stratified by two anatomical levels (L3 and T12) and the global mean across all levels. The visual comparison confirms that the dispersion at the thoracic level (T12) is comparable to that at the standard lumbar level (L3), with compact IQRs across all classes, validating stability across levels. In addition, an analysis of the error distribution was conducted, analyzing the global mean to assess the reliability of the tool based on a high-performance benchmark of DSC≥0.90. While a DSC > 0.80 is often cited as indicating good performance [11], a stricter threshold of 0.90 was selected to ensure optimal segmentation accuracy, consistent with rigorous quality control standards in validation studies [12]. The SM segmentation exhibited robust stability, with 100% (N=518) of cases maintaining a mean DSC≥0.90. Notably, no global segmentation failure (DSC<0.90) was recorded for the SM class. Regarding adipose tissues, the vast majority of cases also achieved high segmentation quality (DSC≥0.90): 99.4% (N=515) and 93.6% (N=485) for global SAT and VAT, respectively. However, unlike the muscle class, a minor subset of the population was identified in the range DSC<0.90 for SAT (0.6%, N=3) and VAT (6.4%, N=33). To further characterize these specific VAT outliers (N=33), a quantitative sub-analysis reveals that geometric failures are exclusively concentrated in patients with low-to-normal adiposity; notably, zero failures occurred in the “Obesity” category (BMI $>30\;{\mathrm{kg}/m}^{2}$). Instead, 91.6% of failures with known metadata were identified in the “Underweight” and “Normal Weight” cohorts. Within these groups, the absolute minimum DSC observed (0.760) corresponded to an extreme underweight case (BMI = $12.7\;{\mathrm{kg}/m}^{2}$). This quantitative distribution confirms our qualitative observations (Figure 4): the scarcity of intra-abdominal adipose tissue in lean and younger patients creates challenging, low-contrast interfaces with adjacent bowel loops, which represent the primary source of geometric error for the model.

Finally, to contextualize the clinical impact of the geometric outliers identified in Figure 3 (cases with DSC <0.90), we conducted a qualitative failure analysis of representative worst-case scenarios. Figure 4 illustrates the segmentation error maps for the patients yielding the highest discrepancies, such as a L3 VAT case with a DSC of 0.738 (BMI = $12.7\;{\mathrm{kg}/m}^{2}$) and a T12 SAT case with a DSC of 0.875 (BMI = $14.4\;{\mathrm{kg}/m}^{2}$). In these maps, green represents correct prediction, red indicates over-segmentation, and blue denotes under-segmentation. Visual inspection reveals that errors are predominantly confined to the complex interfaces between bowel loops and visceral fat. However, even in these suboptimal cases, the system successfully preserves the global anatomical topology, preventing segmentation failures.

### 3.3. Clinical Measurement Precision

To validate the algorithm’s utility for quantitative biomarker extraction, clinical measurement accuracy was assessed by calculating the absolute percentage difference in tissue area (cm^2^) between automated predictions and the expert ground truth (Table 3). It is important to note that this analysis was performed on 2D cross-sectional areas rather than extrapolated 3D volumes. This conservative approach prevents volumetric error cancellation, ensuring that local over-segmentation does not mask under-segmentation in adjacent slices.

The third lumbar vertebra serves as the gold standard for body composition assessment. At this level, the FocusedON-BC framework demonstrated high agreement with ground truth manual segmentation. The MAE (Table 3) remained consistently below the pre-defined performance goal of 5% for all tissue compartments. This strict target was set to ensure that the automated measurement error falls within the typical range of human inter-observer variability reported in the literature [12,14].

For the assessment of sarcopenia, the SM area showed a deviation of only 1.34% (absolute difference: $1.9\;{\mathrm{cm}}^{2}$). Regarding adiposity, SAT quantification yielded an MAE of 0.87% ($1.2\;{\mathrm{cm}}^{2}$). The VAT compartment, which presents greater segmentation challenges due to bowel motion and complex boundaries, maintained a clinically reliable accuracy with an MAE of 4.38% ($3.6\;{\mathrm{cm}}^{2}$).

Recognizing the growing importance of chest CTs for opportunistic screening, the T12 vertebral level was also validated (Table 3). The system maintained robust performance in this upper abdominal region, obtaining an MAE of 2.47% ($2.2\;{\mathrm{cm}}^{2}$) for the SM class, while VAT and SAT demonstrated errors of 4.07% ($2.0\;{\mathrm{cm}}^{2}$) and 1.30% ($0.8\;{\mathrm{cm}}^{2}$), respectively. These results confirm that the algorithm provides reliable quantitative data across the abdominal-thoracic transition zone without introducing systematic errors, expanding its applicability beyond standard abdominal scans.

### 3.4. Algorithm Robustness and Technical Interoperability

To verify that the algorithm maintains consistent performance across diverse clinical and technical scenarios, a stratified analysis was conducted using the validation cohort. The cohort was segmented based on five key variables: sex, BMI, age, CT contrast, and CT manufacturer. The quantitative results of this analysis are consolidated in Table 4. Furthermore, it is important to note that the subgroup lacking demographic metadata (e.g., ‘Unknown BMI’, representing 51.0% of the validation cohort) achieved a mean DSC of 0.978 for SM, 0.965 for VAT, and 0.988 for SAT (Table 4). These metrics are perfectly aligned with those observed in the known BMI categories, confirming that the absence of metadata in real-world retrospective repositories does not mask any systematic failures or compromise the framework’s overall subgroup reliability.

#### 3.4.1. Impact of Sex

Given the distinct anatomical patterns of fat distribution and muscle mass associated with biological sex, we evaluated the model’s performance independently for male and female patients (Table 4). The framework demonstrated expert-level consistency across both cohorts. SM segmentation accuracy was excellent in both males (DSC: 0.976±0.011) and females (DSC: 0.970±0.010). While VAT segmentation showed a slight performance increase in male patients (0.965 vs. 0.950), SAT segmentation proved exceptionally precise in the female cohort, achieving a DSC of 0.989±0.009. Overall, the lowest observed scores remained within clinically acceptable ranges for both sexes, confirming that the tool is reliable for broad population screening regardless of sex-related phenotypic differences.

#### 3.4.2. Impact of Body Mass Index

**Figure 5:**
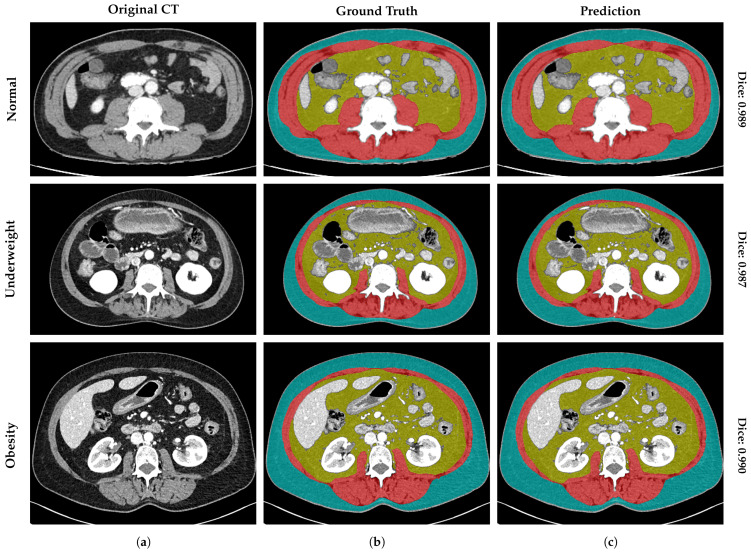
Qualitative segmentation results across different patient profiles. Rows represent three distinct BMI categories: normal weight, underweight, and obesity. (**a**) The original CT image at L3 level, (**b**) the expert manual ground truth, and (**c**) the prediction generated by FocusedON-BC. The vertical labels on the right show the mean dice similarity coefficient (DSC) for each landmark. Red indicates skeletal muscle (SM); yellow indicates visceral adipose tissue (VAT); blue indicates subcutaneous adipose tissue (SAT); and green represents the body region mask.

The system’s performance was evaluated across the entire spectrum of nutritional status, from underweight (<18.5 kg/m^2^) to obesity (>30 kg/m^2^) patients. Figure 5 displays the representative segmentation of these phenotypes, demonstrating the model’s remarkable adaptability to extreme anatomical variations.

As detailed in Table 4, for adipose tissues (VAT and SAT), higher BMI values were associated with better segmentation performance. The “Obesity” cohort ($\mathrm{BMI}>30\;{\mathrm{kg}/m}^{2}$) yielded the highest stability (Figure 5, bottom row), with Mean DSC reaching 0.974±0.015 for VAT and 0.989±0.011 for SAT. In this subgroup, even the worst-case scenarios remained within the pre-defined range (DSC>0.90), confirming that the abundance of adipose tissue facilitates boundary delineation. In contrast, the “Underweight” cohort ($\mathrm{BMI}<18.5\;{\mathrm{kg}/m}^{2}$) represented the most challenging biological scenario due to tissue scarcity. As shown in the middle row of Figure 5, these patients present minimal subcutaneous and visceral fat layers, pushing the limits of spatial resolution. Statistically, the lowest observed scores for VAT (0.760) and SAT (0.859) were observed in this category. Furthermore, the SM compartment demonstrated robust stability against BMI variations. Unlike adipose tissue, SM segmentation quality remained consistent across categories, ranging from a mean DSC of 0.960±0.014 in underweight patients to 0.972±0.008 in obesity patients. Most importantly, no SM segmentation failure (DSC<0.90) was recorded even in the underweight cohort (minimum observed: 0.919), validating the tool’s reliability for sarcopenia assessment in cachectic populations.

#### 3.4.3. Impact of Age

The analysis of age-related anatomical changes demonstrated high stability across the lifespan, from young adulthood to the very elderly (Table 4). The “Very Elderly” cohort demographic is the primary target for sarcopenia assessment but presents specific radiological challenges due to muscle atrophy and myosteatosis. Despite these complexities, the framework demonstrated robust accuracy for SM in this group (mean DSC=0.968±0.011), with no segmentation failures recorded (minimum DSC=0.925). Furthermore, the assessment of adipose compartments in the “Very Elderly” cohort showed consistent performance, suggesting that age-related morphological alterations, such as skin laxity or fat redistribution, did not negatively impact model inference. The model achieved a mean DSC of 0.967±0.021 for VAT and 0.983±0.018 for SAT. Notably, the minimum observed score for VAT in this group was 0.898, bordering the strict pre-defined threshold, which suggests that the algorithm effectively handles the complex visceral texture often found in geriatric patients.

Regarding the “Young Adult” cohort (N=52), this group exhibited the lowest minimum scores for VAT (0.760) and SAT (0.859). Similar to the underweight phenotype, younger individuals frequently exhibit lower volumes of visceral fat, which reduces the distinctness of fascial boundaries. However, even in this subgroup, the mean performance remained high (>0.94 for all tissues), ensuring reliable body composition profiling in early adulthood.

#### 3.4.4. Impact of CT Contrast

To assess the framework’s performance across different imaging protocols, we evaluated the impact of contrast media administration (contrast-enhanced vs. non-contrast scans). As shown in Table 4, the model demonstrated high robustness regardless of the contrast phase. For SM, the mean DSC was 0.973±0.010 in contrast-enhanced scans compared to 0.977±0.010 in non-contrast scans. Similarly, adipose tissue segmentation remained stable, with VAT achieving DSCs of 0.958±0.031 and 0.963±0.040, and SAT achieving 0.986±0.014 and 0.986±0.017 for contrast and non-contrast groups, respectively. The absence of a performance drop in non-contrast images is particularly relevant for clinical applications where contrast may be contraindicated, ensuring the model’s reliability for standardized body composition assessment in diverse clinical trial settings.

#### 3.4.5. Technical Interoperability (Scanner Manufacturer)

Finally, to confirm the vendor neutrality of the system, performance was stratified by CT scanner manufacturer (Table 4). The results indicate minimal variation in SM segmentation, with mean DSC ranging tightly between 0.971±0.009 (Philips) and 0.975±0.009 (Toshiba). Regarding the adipose compartments, the framework demonstrated high adaptability to the varying noise profiles characteristic of each manufacturer. For VAT, which is typically the most sensitive to image texture, the highest mean accuracy was achieved in the smaller cohorts of Toshiba (0.964±0.033, *N* = 48) and Philips (0.962±0.030, *N* = 45), exceeding the dominant Siemens cohort (0.949±0.041, *N* = 292). It is worth noting that, while the absolute worst-case for VAT (DSC=0.760) occurred using a Philips scanner, the high mean performance of this group suggests this was an isolated anatomical outlier. SAT exhibited the highest degree of cross-vendor consistency. The mean DSC was highly comparable across all platforms, ranging only from 0.984 (Toshiba) to 0.986 (Siemens/GE Medical Systems). This demonstrates that detection of the external body contour and the fascia is robust across acquisition devices.

### 3.5. Independent Hold-Out Test

To demonstrate the real-world generalizability of the FocusedON-BC framework beyond the 10-fold cross-validation, a final performance evaluation was conducted on a strictly isolated hold-out dataset. This cohort consisted of 70 unique patients (138 distinct CT slices) completely unseen during the training phase.

As summarized in Table 5, the algorithm successfully replicated the high performance observed during cross-validation across both evaluated landmarks. The spatial segmentation accuracy exceeded the pre-defined clinical safety threshold of 0.90 across all tissue compartments, peaking at the L3 level with a mean DSC of 0.979±0.014 for SM, 0.950±0.041 for VAT, and 0.982±0.032 for SAT. Regarding clinical measurement precision, the absolute volumetric deviations remained strictly bounded. The MAE for the SM and SAT compartments was consistently below 5% at both L3 and T12 landmarks. While the relative percentage error for VAT marginally exceeded this threshold (7.24% at L3 and 7.18% at T12), the absolute geometric deviation remained clinically negligible (e.g., $5.2\;{\mathrm{cm}}^{2}$ at L3). As demonstrated in the cross-validation error analysis, this localized increase in relative error is a recognized mathematical artifact, isolated to patients with extreme visceral fat depletion, in which near-zero ground-truth areas artificially inflate percentage metrics without compromising the actual spatial delineation.

**Figure 6:**
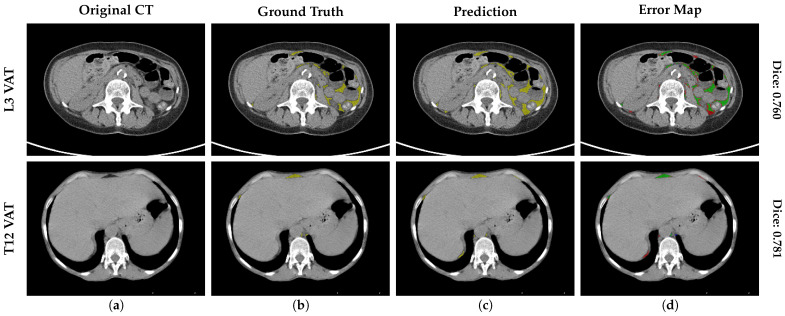
Analysis of worst-case scenarios of the independent hold-out test. Segmentation results for the outliers with the lowest geometric accuracy (DSC < 0.90). (**a**) The original CT image, (**b**) the ground truth mask (VAT in yellow), (**c**) the Prediction (VAT in yellow), and (**d**) the error map highlighting discrepancies, where green pixels represent the correct intersection (true positives), red pixels indicate over-segmentation (false positives), and blue pixels denote under-segmentation (false negatives).

To further contextualize the relative error deviation observed in the VAT compartment, a targeted case-by-case analysis was performed. We confirmed that the elevated percentage MAE (>7%) was almost entirely driven by extreme anatomical outliers characterized by severe physiological fat depletion. This phenomenon is qualitatively illustrated in the error maps provided in Figure 6, where, despite the high relative error, the prediction maintains the global anatomical topology. For instance, the patient exhibiting the highest relative error at L3 (56.3%) was severely underweight ($\mathrm{BMI}=17.6\;\mathrm{kg}/m^{2}$) with a true VAT area of only $7.4\;{\mathrm{cm}}^{2}$. In this scenario, a low absolute segmentation deviation of just $4.1\;{\mathrm{cm}}^{2}$ resulted in a disproportionately inflated mathematical penalty (upper panel in Figure 6). Similarly, at the T12 level (lower panel in Figure 6), extreme cases included deviations as minimal as $0.5\;{\mathrm{cm}}^{2}$ transforming into relative errors exceeding 35% due to the near-zero ground truth denominator ($\mathrm{VAT}=1.5\;{\mathrm{cm}}^{2}$, $\mathrm{BMI}=18.5\;\mathrm{kg}/m^{2}$). Finally, due to the limited sample size of the hold-out cohort, which lacks the statistical power required for reliable subgroup stratification (e.g., specific BMI categories or scanner manufacturers), the detailed technical and clinical robustness analyses remain evaluated exclusively on the fully powered cross-validation dataset.

### 3.6. Qualitative Assessment

Beyond geometric segmentation, a critical feature of FocusedON-BC is its ability to characterize muscle quality by isolating IMAT from contractile lean muscle. As described in the post-processing methodology, this separation is achieved by applying a smooth filter and radiodensity thresholding strictly within the deep fascia envelope predicted by the neural network. This approach, coupled with morphological regularization, ensures biological structural consistency by removing isolated noise artifacts.

**Figure 7:**
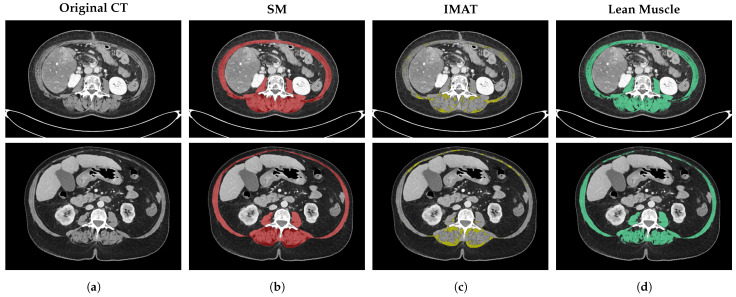
Qualitative assessment of skeletal muscle radiodensity and myosteatosis characterization. (**a**) The original CT image at L3 level; (**b**) the total skeletal muscle boundary predicted by FocusedON-BC in red; (**c**) the intermuscular adipose tissue (IMAT) mask extracted via radiodensity thresholding (<−30 HU) in yellow; and (**d**) the resulting lean-muscle mask (>=−30 HU) in green.

Figure 7 visualizes this decomposition, where the system effectively delineates the outer fascial boundary, preventing the intrusion of subcutaneous fat into the muscle compartment. As shown in the visual qualitative assessment, the tool effectively distinguishes IMAT from contractile lean muscle, enabling the identification of patients with severe myosteatosis, in which the muscle architecture is heavily compromised by low-attenuation adipose tissue.

## 4. Discussion

This study provides a robust multicenter validation of FocusedON-BC, an automated deep learning framework for CT-based body composition assessment. Our results demonstrate that the system can quantify prognostic biomarkers from routine CT scans with expert-level geometric agreement and maintain its accuracy across diverse clinical phenotypes and CT manufacturers. The system demonstrated robust performance that exceeded the strict thresholds defined for this study. Regarding quantitative accuracy, the results strictly adhered to the goal of <5% MAE, confirming reliability within the range of human variability. A DSC above 0.90 is widely accepted as indicative of expert-level performance in medical image segmentation, and the consistently higher values observed in our study suggest minimal geometric deviation from manual reference standards [12,19]. Our results not only meet this standard but position FocusedON-BC competitively against recent state-of-the-art models in the literature. For instance, recent high-performing deep learning frameworks for body composition report DSCs ranging from 0.95 to 0.97 for skeletal muscle and adipose tissues [17,19]. FocusedON-BC’s performance achieves mean DSCs of 0.974 (SM), 0.959 (VAT), and 0.986 (SAT) in the extensive cross-validation cohort (N=518) and strictly maintains these >0.95 optimal levels in the independent hold-out test cohort. These results demonstrate that our fascia-driven architecture equals or exceeds current state-of-the-art benchmarks. Crucially, this expert-level accuracy is sustained across extreme BMI categories and highly heterogeneous, multi-vendor datasets. Previous studies have associated CT-derived muscle and adipose tissue phenotypes with clinically meaningful outcomes, including postoperative complications and mortality. In this context, an automated, reproducible segmentation system could facilitate large-scale opportunistic screening and integration of body composition analysis into routine workflows.

A key strength of our methodology is the accurate delineation of the SM compartment using a fascia-based definition. Unlike many existing algorithms that rely solely on HU thresholds without anatomical boundaries, our approach utilizes deep learning to delineate the SM fascia first. This guarantees that the assessment of muscle quality is restricted to the muscle compartment, effectively excluding surrounding subcutaneous fat. Consequently, we prioritized the validation of this anatomical container. The high accuracy obtained for the SM compartment (DSC=0.974), consistent with recent studies [11,12] combined with the high clinical measurement fidelity (MAE<1.4%), demonstrates that this anatomy-driven strategy effectively excludes surrounding tissues. Unlike pixel-wise classification models that struggle with ambiguous boundaries [13].

A major barrier to the widespread adoption of artificial intelligence in CT-based body composition analysis is the lack of generalization. Algorithms trained on homogeneous datasets often fail when applied to patients with extreme BMI or images from different CT manufacturers. Our study directly addressed this by validating the model in a heterogeneous cohort including underweight (<18.5 ${\mathrm{kg}/m}^{2}$) and obese (>30 ${\mathrm{kg}/m}^{2}$) patients. In underweight patients, where the scarcity of adipose tissue makes the boundaries indistinct, the model maintained DSCs of 0.92 for VAT and 0.96 for SAT. Similarly, in patients with obesity, the performance yielded DSCs of 0.97 for VAT and 0.98 for SAT. Furthermore, the minimal performance variation observed across Siemens, GE, Philips, and Toshiba scanners confirms the tool’s vendor-neutrality. This interoperability is essential for multicenter clinical trials and forms the basis of opportunistic screening. Unlike in controlled research settings, large-scale screening exposes algorithms to unpredictable variations in image quality and acquisition protocols. The demonstrated stability of FocusedON-BC across four major vendors confirms its readiness for deployment in uncurated, real-world clinical data lakes. Parallel to these technical requirements, the stringent regulatory pathway for medical device software. Regulatory frameworks require exhaustive proof of clinical safety, consistency, and generalizability across uncurated populations before a tool can be approved for hospital integration. By strictly validating FocusedON-BC across extreme nutritional phenotypes and multiple CT manufacturers, this study provides the critical clinical evaluation required to meet these regulatory standards. Consequently, this framework bridges the gap between academic research and clinical application, offering a commercially viable, vendor-agnostic solution ready for safe deployment in opportunistic screening workflows. Furthermore, successful opportunistic screening requires computational feasibility. FocusedON-BC was developed as a cloud-native architecture to facilitate seamless clinical integration. The models were highly optimized to run efficiently on standard cloud servers, eliminating the dependency on local, high-performance hardware. This lightweight footprint enables dynamic horizontal scaling, allowing the system to process high volumes of scans concurrently in the cloud without burdening routine hospital IT infrastructures.

Crucially, while our validation focused on standard landmarks (L3, T12), FocusedON-BC is trained to segment any axial level within the T12–L5 range. This flexibility is particularly advantageous for opportunistic screening: if the standard L3 slice is unusable due to metallic artifacts or herniation, the system can successfully process an alternative adjacent slice (e.g., L2 or L4) to retrieve valid body composition metrics, ensuring no patient is excluded due to local image corruption. Although this strict 2D cross-sectional approach directly aligns with current clinical guidelines and prevents the error cancellation phenomenon often observed in volume averaging, we acknowledge the growing relevance of true 3D volumetric analysis. Given the model’s inherent capability to process continuous slices across the abdominal region, future iterations of FocusedON-BC will aim to expand into full 3D volumetric segmentation. This evolution will provide additional value for advanced whole-organ radiomics and the longitudinal tracking of subtle body composition shifts.

Previous studies utilizing the FocusedON-BC framework have already demonstrated strong associations between its derived body composition metrics and clinical prognosis [25,26]. Consequently, the present study was specifically designed to rigorously validate the technical robustness of its underlying segmentation process across diverse data and CT manufacturers. Therefore, it was not designed to correlate the automated body composition metrics with new clinical endpoints (e.g., survival or post-operative complications). While the use of three independent retrospective cohorts effectively mitigated selection bias and provided robust technical validation, future research should now focus on its potential clinical applications: evaluating this framework in real-time clinical settings and facilitating the development of definitive prognostic cut-off values for routine malnutrition screening. Furthermore, to achieve a fully autonomous workflow, the next phase of development will incorporate a vertebral level localization module. This upgrade will enable the system to automatically identify the L3 landmark directly from volumetric CT data, eliminating the need for manual slice selection and streamlining the pipeline for seamless clinical integration.

## 5. Conclusions

FocusedON-BC provides a robust and vendor-agnostic solution for CT-based body composition analysis, achieving expert-level accuracy across diverse nutritional phenotypes and CT manufacturers. By utilizing a fascia-driven segmentation approach, the system effectively overcomes the limitations of traditional radiodensity-based methods, ensuring precise tissue quantification even in challenging scenarios such as sarcopenia or obesity. Given the established clinical relevance of CT-derived muscle and adipose tissue biomarkers, the next step is to integrate the segmentation into routine workflows. The present multicenter validation provides the methodological robustness required to support this transition. Furthermore, FocusedON-BC’s ability to process any axial level within the abdominopelvic volume offers the flexibility necessary for large-scale opportunistic screening, even when standard anatomical landmarks are compromised. Together, these findings support the immediate implementation of our framework in clinical practice, enabling the prognostic value of body composition biomarkers to be realized without delay.

## Figures and Tables

**Table 1:** Demographic, clinical, and CT scanner characteristics of the study populations.

Characteristic	Validation Cohort (N=518)	Hold-Out Test Cohort (N=70)
Sex, *n* (%)		
Male	247 (47.7%)	42 (60.0%)
Female	195 (37.6%)	28 (40.0%)
Unknown	76 (14.7%)	0 (0.0%)
Age (years)		
Mean ± SD	60.8±14.2	63.4±11.9
Age Categories, *n* (%)		
Young Adult (18–44)	52 (10.0%)	5 (7.1%)
Adult (45–64)	193 (37.0%)	30 (42.9%)
Senior (65–79)	165 (32.0%)	31 (44.3%)
Very Elderly (≥80)	32 (6.0%)	4 (5.7%)
Unknown	76 (15.0%)	0 (0.0%)
BMI (${\mathrm{kg}/m}^{2}$)		
Mean ± SD	25.7±5.2	26.4±5.9
BMI Categories, *n* (%)		
Underweight (<18.5)	21 (4.0%)	3 (4.3%)
Normal (18.5–25)	101 (19.5%)	28 (40.0%)
Overweight (25–30)	88 (17.0%)	22 (31.4%)
Obesity (>30)	44 (8.5%)	17 (24.3%)
Unknown	264 (51.0%)	0 (0.0%)
CT Contrast, *n* (%)		
Contrast-enhanced	420 (81.1%)	42 (60.0%)
Non-contrast	98 (18.9%)	28 (40.0%)
CT Manufacturer, *n* (%)		
Siemens Healthineers (Erlangen, Germany)	292 (56.4%)	58 (82.9%)
Philips (Amsterdam, The Netherlands)	65 (12.5%)	0 (0.0%)
GE Medical Systems (Chicago, IL, USA)	53 (10.2%)	10 (14.3%)
Toshiba (Minato, Tokyo, Japan)	48 (9.3%)	2 (2.9%)
Canon Medical Systems (Otawara, Tochigi, Japan)	1 (0.2%)	0 (0.0%)
Unknown	59 (11.4%)	0 (0.0%)

*Note:* SD = Standard Deviation; BMI = Body Mass Index.

**Table 2:** Segmentation performance (Dice similarity coefficient) stratified by vertebral level. Data represents mean ± standard deviation (SD).

Anatomical Level	Dice Similarity Coefficient (Mean ± SD)			
	Body Region	SM	VAT	SAT
Global (All Levels)	0.999±0.001	0.974±0.010	0.959±0.033	0.986±0.014
T12 (Thoracic)	1.000±0.001	0.959±0.016	0.954±0.029	0.976±0.024
L1	0.999±0.001	0.957±0.019	0.951±0.037	0.978±0.029
L2	0.999±0.002	0.976±0.012	0.956±0.036	0.984±0.016
L3 (Standard)	0.999±0.001	0.979±0.009	0.956±0.040	0.988±0.009
L4	0.999±0.001	0.976±0.010	0.954±0.040	0.989±0.008
L5 (Pelvic)	0.999±0.001	0.970±0.013	0.953±0.039	0.990±0.008

Note: SD = Standard Deviation; SM = Skeletal Muscle; VAT = Visceral Adipose Tissue; SAT = Subcutaneous Adipose Tissue.

**Table 3:** Quantitative accuracy of tissue area measurements compared to expert ground truth at L3 and T12 vertebral levels. Data is presented as mean ± standard deviation (SD).

Anatomical Level	Tissue Compartment	MAE (${\mathrm{cm}}^{2}$) ± SD	MAE (%)
L3 (Lumbar)	SM	1.9 ± 1.9	1.34%
	VAT	3.6 ± 3.8	4.38%
	SAT	1.2 ± 1.5	0.87%
T12 (Thoracic)	SM	2.2 ± 2.2	2.47%
	VAT	2.0 ± 2.4	4.07%
	SAT	0.8 ± 1.0	1.30%

Note: MAE = Mean Absolute Error; SM = Skeletal Muscle; VAT = Visceral Adipose Tissue; SAT = Subcutaneous Adipose Tissue.

**Table 4:** Robustness analysis performed using the dice similarity coefficient, which was stratified by sex, BMI, age, CT contrast, and CT manufacturer. Data represents mean ± standard deviation (SD) and the minimum (Min) score observed in the validation cohort.

Subgroup	SM		VAT		SAT	
	Mean ± SD	Min	Mean ± SD	Min	Mean ± SD	Min
Sex						
Male	0.976±0.011	0.919	0.965±0.031	0.760	0.982±0.018	0.817
Female	0.970±0.010	0.925	0.950±0.034	0.827	0.989±0.009	0.923
Unknown Sex	0.977±0.007	0.958	0.962±0.028	0.885	0.990±0.006	0.973
BMI (kg/m^2^)						
Underweight (<18.5)	0.960±0.014	0.919	0.921±0.054	0.760	0.966±0.031	0.859
Normal (18.5–25)	0.969±0.009	0.940	0.939±0.041	0.811	0.981±0.020	0.817
Overweight (25–30)	0.971±0.009	0.925	0.964±0.024	0.831	0.987±0.012	0.889
Obesity (>30)	0.972±0.008	0.950	0.974±0.015	0.910	0.989±0.011	0.923
Unknown BMI	0.978±0.009	0.921	0.965±0.025	0.814	0.988±0.007	0.937
Age Group (Years)						
Young Adult (18–44)	0.974±0.012	0.919	0.941±0.045	0.760	0.982±0.022	0.859
Adult (45–64)	0.975±0.010	0.940	0.956±0.037	0.811	0.986±0.016	0.817
Senior (65–79)	0.973±0.011	0.921	0.965±0.023	0.831	0.986±0.010	0.923
Very Elderly (≥80)	0.968±0.012	0.925	0.967±0.021	0.898	0.983±0.018	0.889
Unknown Age	0.977±0.007	0.958	0.962±0.027	0.885	0.990±0.006	0.973
CT Contrast						
Contrast-enhanced	0.973±0.010	0.921	0.958±0.031	0.811	0.986±0.014	0.817
Non-contrast	0.977±0.010	0.919	0.963±0.040	0.760	0.986±0.017	0.859
CT Manufacturer						
Siemens Healthineers	0.974±0.011	0.919	0.949±0.041	0.827	0.986±0.014	0.859
Philips	0.971±0.009	0.942	0.962±0.030	0.760	0.985±0.009	0.952
GE Medical Systems	0.973±0.012	0.925	0.952±0.035	0.814	0.986±0.008	0.955
Toshiba	0.975±0.009	0.940	0.964±0.033	0.842	0.984±0.028	0.817
Other/Unknown	0.976±0.008	0.958	0.958±0.028	0.885	0.988±0.006	0.970

Note: SD = Standard Deviation; VAT = Visceral Adipose Tissue; SAT = Subcutaneous Adipose Tissue; Min = Minimum observed DSC in the validation fold.

**Table 5:** Segmentation performance (Dice similarity coefficient) and quantitative accuracy (mean absolute error) of tissue area measurements compared to expert ground truth at L3 and T12 vertebral levels using the independent hold-out test. Data is presented as mean ± standard deviation (SD).

Anatomical Level	Tissue Compartment	DSC (Mean ± SD)	MAE (${\mathrm{cm}}^{2}$) ± SD	MAE (%)
L3 (Lumbar)	SM	0.979 ± 0.014	1.3 ± 1.5	0.98%
	VAT	0.950 ± 0.041	5.2 ± 3.3	7.24%
	SAT	0.982 ± 0.032	0.8 ± 0.6	1.04%
T12 (Thoracic)	SM	0.951 ± 0.021	4.0 ± 3.1	4.37%
	VAT	0.932 ± 0.058	3.3 ± 2.7	7.18%
	SAT	0.965 ± 0.063	0.9 ± 0.7	2.68%

Note: DSC = Dice Similarity Coefficient; MAE = Mean Absolute Error; SD = Standard Deviation; SM = Skeletal Muscle; VAT = Visceral Adipose Tissue; SAT = Subcutaneous Adipose Tissue.

## Data Availability

The data presented in this study utilize a mixed-data approach. The primary clinical cohort is not publicly available due to strict privacy and ethical restrictions concerning patient confidentiality and institutional policies regarding clinical data sharing. Portions of the data used for validation (Dataset 2) are publicly available from the SAROS dataset (https://doi.org/10.25737/SZ96-ZG60). The images comprising the independent hold-out test cohort (Dataset 3) are publicly available from The Cancer Imaging Archive (TCIA) in the following collections: TCGA-ESCA (https://doi.org/10.7937/K9/TCIA.2016.VPTNRGFY), ACRIN-FLT-Breast (https://doi.org/10.7937/K9/TCIA.2017.ol20zmxg), VAREPOP-APOLLO (https://doi.org/10.7937/ghkn-md15), and ACRIN-NSCLC-FDG-PET (https://doi.org/10.7937/tcia.2019.30ilqfcl).

## Abbreviations

The following abbreviations are used in this manuscript:

BMI	Body Mass Index
CT	Computed Tomography
DSC	Dice Similarity Coefficient
HU	Hounsfield Unit
IMAT	Intermuscular Adipose Tissue
IQR	Interquartile Range
MAE	Mean Absolute Error
SAT	Subcutaneous Adipose Tissue
SD	Standard Deviation
SM	Skeletal Muscle
VAT	Visceral Adipose Tissue
